# Editorial: Exercising body & brain: the effects of physical exercise on brain health

**DOI:** 10.3389/fnhum.2025.1753714

**Published:** 2025-12-16

**Authors:** Vijaya Majumdar

**Affiliations:** Swami Vivekananda Yoga Anusandhana Samsatahana, Bengaluru, India

**Keywords:** physical exercise, brain health, mental wellbeing, neurobiological mechanisms, psychosocial outcomes, cognitive function, emotional resilience

Physical exercise is widely recognized for its physical health benefits, but emerging evidence robustly illustrates its profound positive impact on brain health and mental wellbeing across diverse populations. This Research Topic collates original research and systematic analyses exploring the interplay between physical activity and brain function, focusing on neurobiological mechanisms and psychosocial outcomes that drive cognitive and emotional resilience. An exemplar study by Li et al. demonstrates how an 8-week, 24-form Tai Chi intervention improved sustained and comprehensive attention in Chinese college students, highlighting the necessity of sustained practice for lasting cognitive benefits. Another pivotal study by Ndabi et al. examined physical activity and anxiety during the COVID-19 pandemic in Tanzania, a low-income setting facing disproportionate mental health challenges. Data from 213 adults showed that vigorous physical activity was significantly linked to reduced anxiety, while moderate activity showed weaker effects; walking and sedentary behaviors had no significant association. Canton-Martinez et al. presented timely evidence that even brief aerobic exercise can enhance memory in older Mexican adults. Comparing 4 weeks of aquatic- and land-based exercise, both groups showed significant improvements in verbal and visual memory, while executive function measures remained unchanged. These results suggest memory is highly responsive to aerobic training, possibly due to improved cerebral blood flow and neurotrophic support. The findings are particularly significant, as impaired memory performance is an independent risk factor for the development of Alzheimer's disease. Given that both the interventions produced similar cognitive benefits highlights accessibility and adaptability in exercise-based cognitive care. Another insightful study by Walter et al. addressed the complex intersection of major depressive disorder (MDD), posttraumatic stress disorder (PTSD), and traumatic brain injury (TBI) within the U.S. military population. This secondary analysis of a clinical trial examined surf and hike therapies among active duty service members with MDD, comparing symptom trajectories between those with (*n* = 47) and without (*n* = 48) a history of TBI. Findings revealed that while TBI history did not significantly moderate short-term improvements in depression or PTSD symptoms following exercise interventions, service members without TBI experienced significantly greater PTSD symptom reduction at 3-month follow-up. The results suggest that TBI may blunt longer-term PTSD symptom improvement despite similar immediate gains across groups. The findings underscore that effects of the intervention may vary based on underlying conditions (such as TBI) or intervention duration, pointing to the need for integrating tailored and longer-term activity-based interventions to optimize cognitive rehabilitation strategies. Yuk et al. compared the effects of aerobic and resistance exercise on mood-related symptoms and EEG activity, finding aerobic exercise reduced anxiety and resistance exercise alleviated depression, along with changes in brain wave patterns. compared 6 weeks of aerobic and resistance exercise with a washout period, assessing anxiety and depression symptoms alongside EEG changes. Aerobic exercise significantly reduced anxiety, while resistance exercise targeted depression. EEG analysis showed decreased theta/beta ratio in the left frontal region after aerobic exercise. These findings highlight the need for personalized exercise strategies for mental health and call for further research on exercise-induced brain wave changes. Collectively, these findings emphasize the importance of culturally tailored, vigorous, and moderate physical activity interventions to boost mental health resilience in resource-limited contexts, across the lifespan and diverse populations, reinforcing the global relevance and nuanced application of exercise for mental wellbeing.

This research collection also contributes significantly to mechanistic aspects of physical activity-based interventions. Krishna et al. conducted an EEG study revealing that frontal alpha asymmetry (FAA) correlates with meditation depth in heartfulness meditators, linking enhanced frontal alpha activity to improved emotional regulation. Their findings identify FAA as a promising biomarker for mindfulness and suggest longitudinal multimodal neuroimaging to explore meditation's causal effects on cognitive-emotional outcomes. The authors advocate for longitudinal multimodal neuroimaging studies to clarify the causal influence of sustained meditation on FAA and related cognitive-emotional outcomes and suggest exploring FAA across various meditation styles and real-time neurofeedback to optimize personalized interventions. Wang et al. meta-analyzed 18 studies on Tai Chi Chuan (TCC), showing neural changes in the superior frontal gyrus related to cognitive control and executive function. Despite limitations like small samples, the study highlights the need for larger standardized neuroimaging research. Together, these studies underscore the frontal brain's key role in cognitive and emotional benefits from mind-body practices. Together, these studies underscore the potential of mind-body practices to enhance mental health through frontal neuroplasticity and call for rigorous, longitudinal, and multimodal approaches to deepen mechanistic understanding and optimize individualized interventions. Another featured article by Yang et al. investigated the mediational role of life satisfaction in the association between exercise and depressive symptoms among middle-aged and elderly individuals. This work highlights how psychosocial wellbeing is not only an endpoint of intervention but also a crucial intermediary in the pathway from physical activity to improved mental health. Such evidence aligns with a growing recognition in the field that positive psychological functioning amplifies the protective effects of exercise against depression. This nuanced pathway also foregrounds the necessity of multidomain interventions for optimal neuropsychiatric outcomes.

Global aging and the rise in neurodegenerative and mood disorders highlight the urgent need for scalable, integrative brain health approaches. Physical activity stands out as a cost-effective strategy for both prevention and symptom improvement in at-risk populations. The methodological diversity and rigor in the included studies reflect advancing sophistication in research design, incorporating innovations like digital health monitoring and multidimensional behavioral metrics. This Research Topic offers a comprehensive overview showing how varied physical activities support brain health across the lifespan, linking mechanisms to clinical and public health aims. Future directions emphasize elucidating molecular and neurophysiological mediators, leveraging digital technologies for personalized interventions, and fostering collaborations between behavioral science, clinical practice, and community health.

